# Expression and Functional Analysis of Peptidoglycan Recognition Protein OfPGRP-B in *Ostrinia furnacalis*

**DOI:** 10.3390/insects17060618

**Published:** 2026-06-11

**Authors:** Xinran Jia, Cunxin Hou, Xueyao Li, Lele Zhang, Yinuo Mao, Zengxia Wang

**Affiliations:** 1 College of Resource and Environment, Anhui Science and Technology University, Chuzhou 233100, China; j13935532795@163.com (X.J.); 18555193459@163.com (C.H.); 2 College of Agriculture, Anhui Science and Technology University, Chuzhou 233100, China; l13865359182@163.com (X.L.); 13637220113@163.com (L.Z.); 19155974145@163.com (Y.M.)

**Keywords:** peptidoglycan recognition protein, antibacterial activity, bacterial agglutination, melanization, innate immunity

## Abstract

To elucidate the role of the peptidoglycan recognition protein OfPGRP-B in the innate immunity of *Ostrinia furnacalis*, a recombinant expression system was established using a prokaryotic expression strategy. The recombinant plasmid encoding *OfPGRP-B* was constructed and expressed in *Escherichia coli*, and the target protein was successfully obtained after induction. Purification and Western blot analysis confirmed that the molecular weight of the recombinant protein was consistent with the predicted value. The immune functions of *OfPGRP-B* were further investigated through antibacterial activity assays, bacterial agglutination tests, amidase activity assays, phenoloxidase (PO) cascade activation, and in vivo melanization assays. The results showed that *OfPGRP-B* alone exhibited weak direct antibacterial activity against the tested bacteria but significantly promoted bacterial agglutination and was capable of degrading peptidoglycan derived from *E. coli* and *Staphylococcus aureus*. In the presence of bacterial peptidoglycan, *OfPGRP-B* enhanced the activation of the PO cascade, but had no significant effect on in vivo melanization. These findings suggest that *OfPGRP-B* may function as an immunomodulatory factor involved in the humoral immune response of *O. furnacalis*, providing a theoretical basis for the development of biological control strategies based on host immune mechanisms.

## Simple Summary

This study investigates the peptidoglycan recognition protein OfPGRP-B in *Ostrinia furnacalis*, a pest that impacts maize production. The *OfPGRP-B* gene was successfully expressed in *Escherichia coli*, and a series of functional assays were conducted. Results showed that OfPGRP-B exhibited weak antibacterial effects but significantly promoted bacterial agglutination and displayed amidase activity. It also enhanced the activation of the prophenoloxidase cascade in the presence of bacterial peptidoglycan but did not affect melanization. These findings suggest that *OfPGRP-B* plays an essential role in the immune response of *Ostrinia furnacalis* and could provide insights for developing biological control strategies targeting host immune mechanisms.

## 1. Introduction

The *Asian corn borer*, *Ostrinia furnacalis* (*Lepidoptera: Crambidae*), is one of the most destructive stem-boring pests affecting maize production in China and many other Asian regions, occurring in nearly all major maize-growing regions except the Qinghai–Tibet Plateau [1]. Larvae feed on maize stalks, leaf sheaths, and ears, causing direct damage such as plant lodging and poor grain development. In addition, feeding wounds facilitate the invasion of pathogenic microorganisms, resulting in further declines in both yield and quality [2]. It has been reported that yield losses caused by *O. furnacalis* can reach approximately 10% in normal outbreak years and exceed 30% during severe infestations, posing a persistent threat to food security [3,4].

Chemical pesticides have long been the primary method for controlling *O. furnacalis*. However, their extensive and repeated use has led to a range of issues, including increased insecticide resistance, adverse effects on non-target organisms, and environmental pollution [5]. Under these circumstances, biological control strategies based on entomopathogenic microorganisms have gained increasing attention. Studies have widely reported the application of *Bacillus thuringiensis* for the control of *O. furnacalis*, with its insecticidal activity mainly depending on Cry toxins that disrupt the larval midgut epithelium [1,6,7,8]. In addition, the entomopathogenic fungus *Beauveria bassiana* has also been extensively used as a microbial control agent against *O. furnacalis*, infecting insects through cuticle penetration and suppression of host immune responses [9,10]. Nevertheless, the efficacy of microbial control agents depends not only on their intrinsic virulence but also on the immune response of the host insect [11]. Therefore, elucidating the mechanisms by which *O. furnacalis* recognizes and responds to pathogenic microorganisms is essential for improving the effectiveness of biological control.

Unlike vertebrates, insects lack adaptive immunity and rely primarily on innate immune systems to defend against pathogen invasion [12]. Pattern recognition receptors (PRRs) play a central role in recognizing pathogen-associated molecular patterns (PAMPs). Among them, peptidoglycan recognition proteins (PGRPs) are key PRRs that specifically recognize bacterial peptidoglycan and are widely distributed in insects and other invertebrates [13]. In *Drosophila*, PGRP-LC and PGRP-LE recognize peptidoglycan from Gram-negative bacteria, thereby activating the immune deficiency (IMD) signaling pathway and inducing antimicrobial peptide expression. These mechanisms have become classical models for studying antibacterial immunity in insects [14,15]. In addition, PGRPs exhibit functional diversification; some members not only participate in immune signaling but also possess amidase activity, enabling them to degrade peptidoglycan directly and thereby regulate immune responses and maintain immune homeostasis [16].

In recent years, the functions of PGRP family members have been increasingly characterized in various agricultural pests. For example, specific PGRPs in *Plutella xylostella*, *Aedes aegypti*, and *Bombyx mori* have been shown to participate in antibacterial defense, regulate gut microbiota homeostasis, and even influence host susceptibility to microbial insecticides [17,18,19]. In contrast, studies on the PGRP family in *O. furnacalis* remain limited, and their specific roles in immune responses have not been systematically elucidated.

Based on previous transcriptomic analyses, we found that the expression of the *OfPGRP-B* gene was significantly upregulated in response to bacterial challenge, suggesting that it may be involved in bacterial recognition and immune signaling. In addition, our previous study systematically analyzed the bioinformatic characteristics of *OfPGRP-B*, including its gene structure, conserved domains, and evolutionary relationships. Therefore, in the present study, we focused on the experimental validation of its biological functions rather than repeating the bioinformatic analyses [20].

However, gene expression data alone are insufficient to fully elucidate the biological role of *OfPGRP-B*, and functional validation at the protein level is therefore necessary. Understanding the immune-related functions of this protein is important for clarifying the molecular mechanisms underlying host immune regulation in *O. furnacalis*. Previous studies have demonstrated that PGRPs participate in insect immunity through multiple mechanisms. First, some PGRPs exhibit direct antibacterial activity, either independently or in cooperation with metal ions [21]. Second, PGRPs can promote bacterial agglutination, thereby limiting pathogen dissemination [11]. Third, amidase-active PGRPs can degrade peptidoglycan and modulate immune signaling input [22]. Finally, PGRPs may also participate in humoral immune processes such as the prophenoloxidase (proPO) cascade and melanization responses [23,24,25].

Therefore, in this study, the recombinant *OfPGRP-B* protein was obtained through prokaryotic expression, and its potential immune functions were systematically investigated. A series of functional assays, including antibacterial activity, bacterial agglutination, amidase activity, proPO cascade activation, and in vivo melanization assays, were conducted to comprehensively elucidate the role of *OfPGRP-B* in the innate immune response of *O. furnacalis*. This work aims to provide a theoretical basis for understanding host–pathogen interactions and the immune functions of *OfPGRP-B* in *O. furnacalis*.

## 2. Materials and Methods

### 2.1. Insect Rearing

Larvae of *O. furnacalis* were obtained from the insect-rearing facility of Anhui Science and Technology University, Chuzhou, China, and maintained under controlled laboratory conditions in an artificial climate chamber. The rearing conditions were as follows: temperature of (28 ± 1) °C, relative humidity of approximately 80%, and a photoperiod of 16 h light and 8 h dark (L:D = 16 h:8 h) [26].

### 2.2. Experimental Materials

The main reagents used in this study included TRIzol^®^ Reagent, ddH_2_O, GoldView nucleic acid stain (10,000×), 50× TAE buffer, and *E. coli* DH5α competent cells (Sangon Biotech, Shanghai, China). The 2× Es Taq SuperMix kit, DNA loading buffer, and DNA marker were purchased from TransGen Biotech (Beijing, China). The bacterial strains used in this study were provided by Beijing Beina Chuanglian Biotechnology Co., Ltd. (Beijing, China). DNA gel purification was performed using the AxyPrep DNA Gel Extraction Kit (Axygen, Union City, CA, USA), and ligation reactions were conducted using the pGEM-T Easy Vector System (Promega, Madison, WI, USA).

### 2.3. Construction of Recombinant Prokaryotic Expression Plasmid

Total RNA was extracted from *O. furnacalis* larvae using TRIzol reagent (Invitrogen, Carlsbad, CA, USA) according to the manufacturer’s instructions. RNA samples from the three biological replicates were reverse-transcribed using the One-Step gDNA Removal and cDNA Synthesis SuperMix kit (TransGen Biotech, Beijing, China) according to the manufacturer’s instructions. The coding sequence of the *OfPGRP-B* gene was amplified by polymerase chain reaction (PCR) using the synthesized cDNA as a template and subsequently used for the construction of the prokaryotic expression vector [27]. The PCR was performed in a 20 μL mixture containing 10 μL of 2× Taq PCR Master Mix (blue dye), 1 μL of forward primer, 1 μL of reverse primer, 1 μL of cDNA template, and 7 μL of ddH_2_O. The amplification conditions consisted of an initial denaturation at 94 °C for 3 min, followed by 30 cycles of 94 °C for 30 s, 60 °C for 30 s, and 72 °C for 30 s, with a final extension at 72 °C for 10 min and storage at 4 °C. The PCR products were analyzed by 1% agarose gel electrophoresis in 1× TAE buffer at 120 V for 30 min and stained with GoldView nucleic acid stain. A single distinct band corresponding to the expected size of the *OfPGRP-B* coding sequence was observed, indicating successful amplification. The PCR products were then purified using the AxyPrep DNA Gel Extraction Kit (Axygen, Union City, CA, USA). The purified fragments were ligated into the pGEM-T Easy Vector System (Promega, Madison, WI, USA) and transformed into *E. coli* DH5α competent cells for plasmid propagation and amplification. Positive clones were screened by colony PCR, and recombinant plasmids were extracted and sent to Sangon Biotech (Shanghai, China) for sequencing verification.

### 2.4. Expression, Purification, and Western Blot Analysis of Recombinant Protein

The verified recombinant plasmid was subsequently transformed into *E. coli* BL21 (DE3) competent cells for recombinant protein expression. A single colony was inoculated into LB liquid medium and cultured overnight at 37 °C with shaking at 220 rpm. Protein expression was induced by adding Isopropyl β-D-thiogalactopyranoside (IPTG) to a final concentration of 0.5 mM, followed by continued incubation for 4 h [28].

After induction, the bacterial culture was centrifuged at 1110× *g* for 10 min to collect the cell pellet, which was then resuspended in buffer. The cells were lysed by ultrasonication. The lysate was centrifuged to separate the supernatant and pellet fractions, which were subsequently analyzed by SDS-PAGE. Protein expression was analyzed by 12% sodium dodecyl sulfate–polyacrylamide gel electrophoresis (SDS-PAGE).

The target protein was subsequently purified using Ni^2+^-NTA metal affinity chromatography. Eluted fractions were collected and dialyzed overnight against phosphate-buffered saline (PBS) buffer using a dialysis membrane. The dialyzed protein samples were analyzed again by SDS-PAGE, and purified proteins were stored at −80 °C until further use. Finally, the recombinant OfPGRP-B protein was specifically identified by Western blot analysis.

### 2.5. Functional Characterization of Recombinant OfPGRP-B Protein

#### 2.5.1. Antibacterial Activity

Three bacterial species, *E. coli*, *B. thuringiensis*, and *S. aureus*, were selected as representative Gram-negative and Gram-positive bacteria commonly used in insect immune studies. The bacteria were cultured in liquid medium at 37 °C with shaking at 200 rpm until reaching an optical density of OD_600_ = 0.6. Reaction mixtures were prepared in 1.5 mL Eppendorf tubes (see Table 1). Bacterial suspensions were mixed with different treatment solutions in Luria–Bertani (LB) medium, in which Zn^2+^ was supplemented in the corresponding treatment group because previous studies demonstrated that the bactericidal activity of PGRPs is Zn^2+^ dependent, with Zn^2+^ primarily functioning as a cofactor facilitating PGRP-mediated antibacterial activity, and incubated in a shaker at 37 °C and 200 rpm for 2 h [29]. After incubation, the bacterial cultures were diluted 10^4^-fold, and 20 μL of each dilution was evenly spread onto solid agar plates. Plates were incubated upside down at 37 °C overnight. BSA was used as a non-specific protein control, whereas PBS served as a blank control. Each treatment was performed in triplicate, and antibacterial activity was evaluated by counting colony-forming units (CFUs).

#### 2.5.2. Bacterial Agglutination Assay of Recombinant Protein

Activated bacteria were inoculated into fresh medium and cultured until the cell density reached OD_600_ = 1.0. Subsequently, 20 μL of bacterial suspension was mixed with 20 μL of recombinant OfPGRP-B protein or BSA (0.5 mg/mL), together with 4 μL of CaCl_2_ (100 mM). Ca^2+^ was included because previous studies have demonstrated that Ca^2+^ facilitates carbohydrate recognition and bacterial binding during lectin-like agglutination processes, whereas Zn^2+^ mainly functions as a catalytic cofactor for amidase activity [30]. The mixture was incubated at 28 °C with shaking at 100 r/min for 1 h [31].

After incubation, 10 μL of the reaction mixture was placed onto a glass slide, and bacterial aggregation was observed using a confocal microscope (SMZ168-TL, Motic, Xiamen, China) to evaluate the agglutination activity of the recombinant protein.

#### 2.5.3. Amidase Activity

Insoluble peptidoglycan (PGN) derived from *E. coli*, *B. thuringiensis*, and *S. aureus* was diluted in PBS to a final concentration of 1 mg/mL. In a 96-well plate, 10 μL of PGN suspension (1 mg/mL) was mixed with either 4 μL of OfPGRP-B protein (200 μg/mL) or 4 μL of BSA (200 μg/mL). The reaction volume was adjusted to 60 μL using PBS containing 15 mM ZnCl_2_. The absorbance at 540 nm was measured at 0, 1, 2, 3, 4, and 5 h using a microplate reader, with shaking prior to each measurement. The degradation of PGN was used to evaluate the amidase activity of the recombinant protein [22,32]. Each treatment was performed with three independent replicates. The absorbance values are presented as the mean ± SD, and statistical differences among treatments were analyzed using one-way ANOVA followed by Tukey’s multiple comparison test.

#### 2.5.4. Prophenoloxidase Cascade

Fifteen fifth-instar larvae on the third day after molting were selected. The larvae were surface-sterilized with 70% ethanol and anesthetized on ice for approximately 15 min. Hemolymph was collected by cutting the proleg and transferred into pre-chilled 1.5 mL Eppendorf tubes. The samples were centrifuged at 16,000× *g* for 30 s at 4 °C to remove hemocytes, and the supernatant, referred to as hemolymph plasma, was collected.

The reaction system was prepared according to Table 2. Hemolymph plasma was mixed with PBS, recombinant OfPGRP-B protein, and bacterial PGN. The reaction mixture was then gently mixed and incubated on ice for 2 min. The mixture was then centrifuged at 10,000× *g* for 1 min. Subsequently, 5 μL of the supernatant was transferred into a 96-well plate, and 100 μL of dopamine solution (2 mM; Macklin, Shanghai, China) was added to each well simultaneously [24]. The absorbance at 490 nm was measured immediately after dopamine addition using a Tecan Infinite 200 Pro microplate reader to monitor the formation of dopachrome, which reflects phenoloxidase activity and was used as an indicator of proPO cascade activation.

#### 2.5.5. Melanization

Sephadex DEAE A-25 beads were washed three times with PBS and resuspended to a concentration of approximately 100–200 beads/μL. A total of 10 μL of bead suspension was incubated with 150 μL of recombinant OfPGRP-B protein (500 μg/mL) and gently mixed, followed by slow shaking at room temperature overnight. BSA and PBS treatments were used as controls.

After incubation, the beads were gently washed six times with PBS to remove unbound protein and resuspended to a concentration of approximately 100–120 beads/μL. Using a microinjector (PDE 0003, Burkard, Rickmansworth, UK), 5 μL of bead suspension was injected into the hemocoel of fifth-instar *O. furnacalis* larvae, with six replicates per group [33]. After injection, the larvae were maintained in an incubator at 28 ± 1 °C and approximately 80% relative humidity for 24 h. The larvae were then dissected, and the beads were recovered. The degree of melanization was evaluated under a stereomicroscope according to the proportion of bead surface covered by melanin, as previously described: level 0, no melanization; level 1, approximately 1–50% melanized; level 2, 50–90% melanized; and level 3, 90–100% melanized. Beads classified as levels 2–3 were considered heavily melanized, and the melanization rate was calculated as the percentage of heavily melanized beads among the total recovered beads [34].

### 2.6. Statistical Analysis

All experimental data were analyzed using SPSS 25.0 software (IBM Corp., Armonk, NY, USA). Gene expression data are presented as the mean ± standard deviation (mean ± SD) based on three biological replicates. Differences among treatment groups were assessed using one-way analysis of variance (ANOVA), followed by Tukey’s multiple comparison test. A *p*-value < 0.05 was considered to indicate statistical significance.

## 3. Results

### 3.1. Expression, Purification, and Identification of Recombinant OfPGRP-B

**Figure 1:**
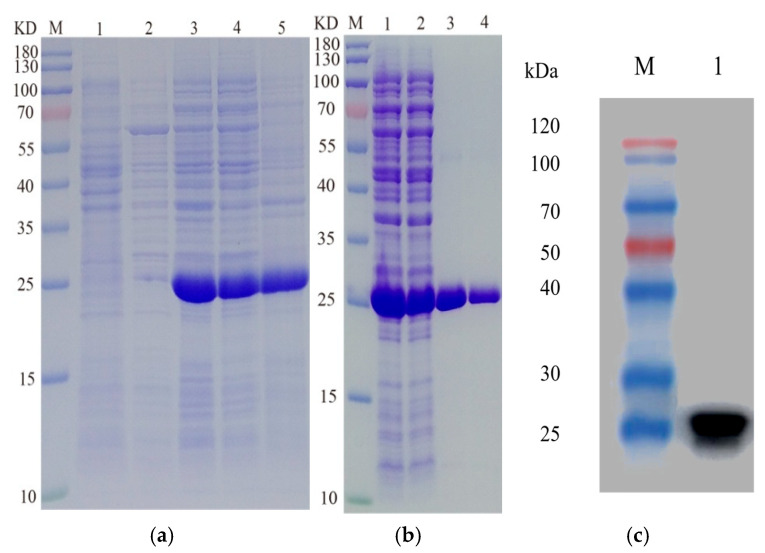
Induced expression of Recombinant protein PGRP-B. Note: (**a**) SDS-PAGE analysis for protein expression identification: M—Protein molecular quality standards, 1—Induced empty pCZN1 vector control (no recombinant protein expression), 2—Not induced, 3—After induction, 4—Supernatant after sonication, 5—Pellet after sonication; (**b**) SDS-PAGE analysis for protein purification: M—Protein molecular quality standards, 1—Crude lysate, 2—Flow-through fraction, 3—Elution fraction 1, 4—Elution fraction 2; (**c**) Protein Western Blot Identification Analysis: M—Protein molecular quality standards, 1—Purified sample.

The recombinant OfPGRP-B protein was successfully expressed after induction, as shown in lane 3 of Figure 1a. The protein was detected in both the supernatant (lane 4) and the pellet (lane 5) after cell lysis, with an apparent molecular weight of approximately 25 kDa. After purification, the recombinant protein appeared as a single distinct band (lanes 3–4 in Figure 1b). Western blot analysis further confirmed the identity of the purified protein, showing a clear and specific band (lane 1 in Figure 1c). These results indicate that the molecular weight of the expressed protein is consistent with the predicted size of the target protein.

### 3.2. Antibacterial Activity Assay

**Figure 2:**
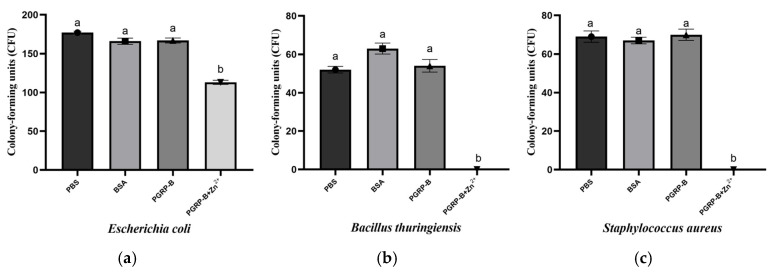
Antibacterial activity of recombinant *OfPGRP-B* against the tested bacteria. Antibacterial activity was evaluated by counting colony-forming units (CFU) after treatment. Data are shown as the mean ± SD from three independent replicates. Different letters indicate significant differences (*p* < 0.05). (**a**) *Escherichia coli*; (**b**) *Bacillus thuringiensis*; (**c**) *Staphylococcus aureus*.

The antibacterial activity of *OfPGRP-B* was evaluated by CFU assays against *E. coli*, *B. thuringiensis*, and *S. aureus* (Figure 2). For *E. coli*, no significant differences in CFU counts were observed among the PBS, BSA, and *OfPGRP-B* groups, with average CFU counts of 177, 161, and 167, respectively (*p* > 0.05). This result indicates that *OfPGRP-B* alone did not exhibit obvious antibacterial activity against *E. coli* under these experimental conditions. Addition of Zn^2+^ significantly reduced the CFU count to 113, representing a 32.3% decrease compared with the *OfPGRP-B* group (*p* < 0.05). For *B. thuringiensis*, no significant differences were observed among the PBS, BSA, and *OfPGRP-B* groups, with average CFU counts of 52, 63, and 54, respectively (*p* > 0.05). In contrast, no colonies were detected after *OfPGRP-B* + Zn^2+^ treatment. A similar trend was observed for *S. aureus*. The PBS, BSA, and *OfPGRP-B* groups showed comparable CFU counts (69, 67, and 70, respectively; *p* > 0.05), whereas no colonies were observed in the *OfPGRP-B* + Zn^2+^ group.

### 3.3. Bacterial Agglutination Assay

**Figure 3:**
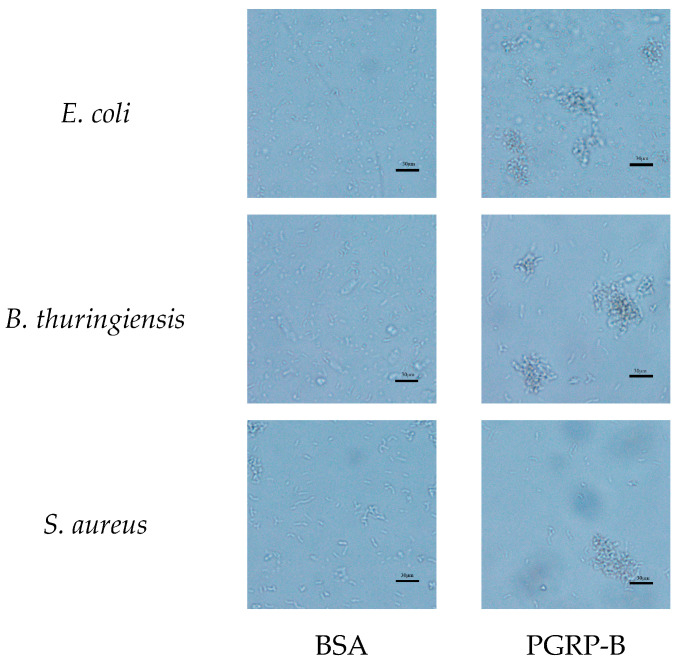
Agglutination of the tested bacteria by recombinant *OfPGRP-B*. BSA was used as the control.

Diluted suspensions of *E. coli*, *B. thuringiensis*, and *S. aureus* were incubated with BSA or recombinant PGRP-B protein, respectively, and bacterial agglutination was observed. As shown in Figure 3, compared with the BSA control, recombinant *OfPGRP-B* induced visible agglutination of *E. coli*, *B. thuringiensis*, and *S. aureus*, with a relatively more pronounced agglutination effect observed for *S. aureus*.

### 3.4. Amidase Activity Assay

**Figure 4:**
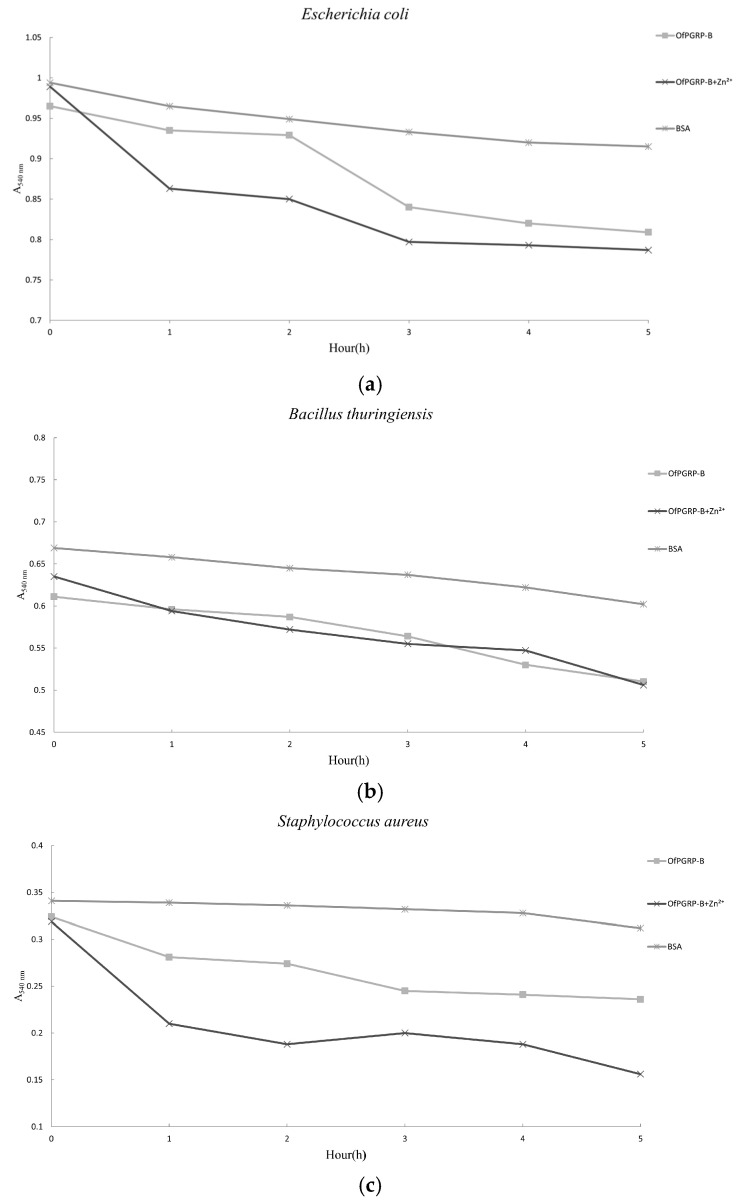
Amidase activity assay of recombinant *OfPGRP-B*. PGN derived from (**a**) *E. coli*, (**b**) *B. thuringiensis*, and (**c**) *S. aureus* was incubated with recombinant *OfPGRP-B*, recombinant *OfPGRP-B* plus Zn^2+^, or BSA. Absorbance was measured at 540 nm and expressed as A_540_.

The amidase activity of recombinant *OfPGRP-B* was evaluated using insoluble PGN from *E. coli*, *B. thuringiensis*, and *S. aureus* as substrates (Figure 4). In the *E. coli* and *S. aureus* groups, the A_540_ values gradually decreased during incubation with *OfPGRP-B*, and a greater decrease was observed after Zn^2+^ addition. After 5 h, the A_540_ values decreased from 0.965 to 0.809 for *E. coli* PGN and from 0.324 to 0.236 for *S. aureus* PGN in the *OfPGRP-B* groups, whereas the *OfPGRP-B* + Zn^2+^ groups decreased further to 0.787 and 0.156, respectively. In contrast, the BSA groups showed only slight changes during incubation. A similar reduction trend was observed for *B. thuringiensis*-derived PGN. The A540 values decreased from 0.611 to 0.510 in the *OfPGRP-B* group and from 0.635 to 0.506 in the *OfPGRP-B* + Zn^2+^ group, while the BSA control remained relatively stable throughout the incubation period.

### 3.5. Prophenoloxidase Cascade Assay

**Figure 5:**
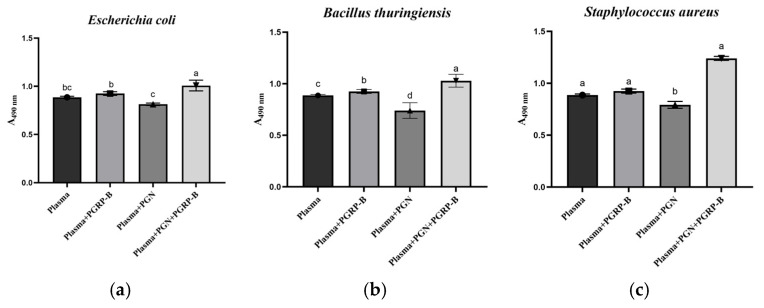
proPO cascade activation assay. Plasma indicates hemolymph plasma, i.e., the supernatant obtained from hemolymph after removal of hemocytes by centrifugation. Dopachrome formation was monitored at 490 nm as an indicator of phenoloxidase activity. Note: (**a**) *E. coli*; (**b**) *B. thuringiensis*; (**c**) *S. aureus*; Different letters indicate significant differences (*p* < 0.05).

As shown in Figure 5, *OfPGRP-B* alone had little effect on PO activity. In contrast, co-incubation with PGN markedly increased PO activity in all three bacterial groups. For *E. coli*, the A_490_ value increased from 0.814 in the Plasma + PGN group to 1.008 in the Plasma + PGN + *OfPGRP-B* group. Similar increases were observed for *B. thuringiensis* and *S. aureus*, with A_490_ values increasing from 0.740 to 1.029 and from 0.792 to 1.240, respectively. The highest A_490_ value was observed in the *S. aureus* group.

### 3.6. Melanization Assay

**Figure 6:**
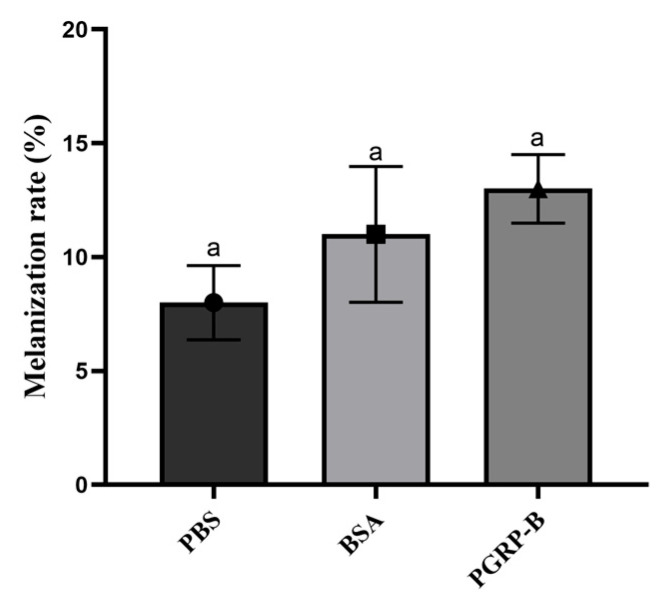
In vivo melanization assay of Sephadex beads. Melanization rate represents the percentage of heavily melanized beads (levels 2–3) among the total recovered beads. Different letters indicate significant differences (*p* < 0.05), whereas bars sharing the same letter are not significantly different (*p* > 0.05).

Sephadex beads pre-incubated with PBS, BSA, or PGRP-B were injected into the hemocoel of *O. furnacalis* larvae. After 24 h, the larvae were dissected, and the beads were recovered to determine the melanization rate. The proportion of heavily melanized beads recovered from larvae injected with *OfPGRP-B*-treated beads was 13%, which was not significantly different from that of the PBS- and BSA-treated control groups (8% and 11%, respectively) (Figure 6).

## 4. Discussion

PGRPs have been widely reported to participate in bacterial recognition and immune regulation in insects. However, the functional properties of *OfPGRP-B* in *O. furnacalis* at the protein level remain insufficiently characterized. In the present study, recombinant *OfPGRP-B* was shown to mediate bacterial agglutination, exhibit peptidoglycan-degrading activity, and enhance PGN-dependent proPO cascade activation, suggesting that *OfPGRP-B* contributes to humoral immune responses in *O. furnacalis* [35].

In this study, based on prior transcriptomic analysis, the *OfPGRP-B* gene, which showed significant responsiveness to bacterial stimulation, was identified. Recombinant *OfPGRP-B* protein was successfully expressed and purified using a prokaryotic expression system, providing a solid foundation for subsequent functional characterization. A series of functional assays were conducted to systematically investigate the potential role of *OfPGRP-B* in the innate immune response of *O. furnacalis*.

In the antibacterial activity assay, *OfPGRP-B* exhibited limited direct inhibitory effects on the tested bacteria in the absence of cofactors. However, its antibacterial activity was markedly enhanced in the presence of Zn^2+^, particularly against *B. thuringiensis* and *S. aureus*. Previous studies have shown that Zn^2+^ is required for the bactericidal activity of PGRPs and primarily acts as a cofactor facilitating PGRP-mediated antibacterial function rather than directly targeting bacteria under physiological conditions. Some amidase-type PGRPs depend on Zn^2+^ to maintain their catalytic conformation, thereby enabling peptidoglycan degradation or immune regulatory functions. Zhang et al. [32] reported in *Plutella xylostella* that short-type PGRPs not only participate in pathogen recognition but also regulate immune signaling intensity through amidase activity. Similarly, Yang et al. [36] demonstrated in mussels that the catalytic activity of amidase-type PGRPs is closely associated with metal ion binding. The Zn^2+^ dependence observed for *OfPGRP-B* in this study suggests that its functional mechanism may be similar to that of previously reported amidase-type PGRPs [12]. These findings further support the potential role of *OfPGRP-B* as a Zn^2+^-dependent immune effector involved in antibacterial defense in *O. furnacalis*.

The bacterial agglutination assay demonstrated that *OfPGRP-B* promoted the aggregation of the three tested bacteria, namely *E. coli*, *B. thuringiensis*, and *S. aureus*. Previous studies suggest that PGRP-mediated bacterial agglutination helps restrict pathogen dissemination and enhances the efficiency of pathogen clearance by the host humoral immune system. Since bacterial agglutination is closely associated with pathogen recognition and surface binding processes, Ca^2+^ was supplemented in the assay because it has been widely reported to facilitate carbohydrate recognition and lectin-like binding interactions, whereas Zn^2+^ mainly functions as a catalytic cofactor for amidase-active PGRPs. Kaneko et al. [31] reported in *Drosophila* that PGRP recognition of bacterial peptidoglycan can induce pathogen aggregation and is closely associated with activation of the Imd pathway. Subsequently, Zhang et al. [32] observed similar phenomena in Lepidopteran insects and proposed that agglutination serves as an auxiliary mechanism in humoral immunity by restricting pathogen dissemination and facilitating pathogen clearance by immune effectors [37]. The ability of *OfPGRP-B* to agglutinate different bacterial species further supports its functional role in the humoral immune defense of *O. furnacalis*.

In the amidase activity assay, *OfPGRP-B* exhibited the ability to degrade peptidoglycan from certain bacterial sources, which is consistent with the functional characteristics of amidase-type PGRPs reported in various insects and invertebrates. It has been suggested that amidase-active PGRPs not only directly degrade pathogen components but also contribute to immune homeostasis by reducing immune signaling input, thereby preventing excessive immune responses [23]. In addition, some PRRs may indirectly promote proPO activation by enhancing pathogen signal input or amplifying immune responses [36]. Based on these findings, *OfPGRP-B* may possess dual functions in both pathogen recognition and immune regulation [38].

The proPO cascade is an important component of insect humoral immunity [24]. In this study, *OfPGRP-B* enhanced proPO cascade activation only when co-present with peptidoglycan, whereas its effect alone was minimal. This suggests that *OfPGRP-B* may function as a cooperative regulator rather than a direct activator in this process.

Unlike the enhanced proPO cascade activation observed in vitro, no significant differences were observed in the in vivo melanization assay [25]. Previous studies have shown that different PGRP members exhibit distinct functional hierarchies in immune responses, with some primarily involved in pathogen recognition or immune homeostasis rather than directly mediating melanization. Song et al. [33] reported in mosquitoes that certain PGRPs regulate host immunity by modulating gut microbiota homeostasis but do not directly participate in melanization. Similarly, Song et al. [18] suggested that the in vivo functions of immune recognition molecules often depend on specific tissue environments and stimulation intensity. The results of this study indicate that *OfPGRP-B* may not be a key regulator of melanization in *O. furnacalis*.

## 5. Conclusions

In this study, the peptidoglycan recognition protein OfPGRP-B from *O. furnacalis* was successfully identified, cloned, and expressed. Expression profiling revealed that *OfPGRP-B* is predominantly expressed in immune-related tissues and is significantly induced upon bacterial challenge, suggesting its involvement in host defense. The recombinant protein was successfully produced in a prokaryotic system, providing a basis for functional characterization. Our results indicate that *OfPGRP-B* likely participates in the recognition of bacterial components and may contribute to the regulation of immune responses in *O. furnacalis*. These findings enhance our understanding of the innate immune functions of *OfPGRP-B* in *O. furnacalis*.

## Figures and Tables

**Table 1:** Antibacterial activity detection reaction system.

Control Group	Experimental Group 1	Experimental Group 2	Experimental Group 3
Test bacterium, 50 μL	Test bacterium, 50 μL	Test bacterium, 50 μL	Test bacterium, 50 μL
LB medium, 100 μL	LB medium, 100 μL	LB medium, 100 μL	LB medium, 100 μL
PBS buffer, 150 μL	PGRP protein solution (0.5 mg/mL), 120 μL	PGRP protein solution (0.5 mg/mL), 120 μL	BSA protein solution (0.5 mg/mL), 120 μL
—	PBS buffer, 30 μL	PBS buffer (containing ZnCl_2_), 30 μL	PBS buffer, 30 μL

**Table 2:** Reaction mixtures for the prophenoloxidase cascade assay.

Control Group	Experimental Group 1	Experimental Group 2	Experimental Group 3
Hemolymph plasma, 5 μL	Hemolymph plasma, 5 μL	Hemolymph plasma, 5 μL	Hemolymph plasma, 5 μL
PBS buffer, 15 μL	PBS buffer, 13 μL	PBS buffer, 13 μL	PBS buffer, 11 μL
—	Recombinant OfPGRP-B protein solution (0.5 mg/mL), 2 μL	Peptidoglycan from test bacterium, 2 μL	Recombinant OfPGRP-B protein solution (0.5 mg/mL), 2 μL
—	—	—	Peptidoglycan from test bacterium, 2 μL

## Data Availability

The original contributions presented in this study are included in the article. Further inquiries can be directed to the corresponding author.
